# miRNA-331-3p Affects the Proliferation, Metastasis, and Invasion of Osteosarcoma through SOCS1/JAK2/STAT3

**DOI:** 10.1155/2022/6459029

**Published:** 2022-09-26

**Authors:** Dan Zu, Qi Dong, Sunfang Chen, Yongde Chen, Jun Yao, Yubin Zou, Jiawei Lin, Bin Fang, Bing Wu

**Affiliations:** ^1^Central Laboratory, The Central Hospital, Shaoxing University, Shaoxing 312030, China; ^2^Department of Spine Surgery, The Central Hospital, Shaoxing University, Shaoxing 312030, China

## Abstract

MicroRNAs (miRNAs) are regulatory small noncoding RNAs that play a key role in several types of cancer. It has been reported that miR-331-3p is involved in the development and progression of various cancers, but there are few reports regarding osteosarcoma (OS). The public GEO database was used to analyze the survival difference of miR-331-3p in OS organizations. The level of cell proliferation assay was assessed by CCK-8 and colony formation. First, transwell and wound-healing assays were used to detect the transfer and invasion ability of miR-331-3p in OS. Second, TargetScan, miRDBmiR, TarBase, and dual-luciferase reporter gene assays were used to determine SOCS1 as a targeted regulator. Third, Western blot and immunohistochemistry were used to detect the expression of protein levels. Finally, a mouse model of subcutaneously transplantable tumors is used to evaluate the proliferation of OS in vivo. The low expression of miR-331-3p was negatively correlated with the overall survival of OS patients. Overexpression of miR-331-3p significantly inhibited cell proliferation, metastasis, and invasion. Moreover, miR-331-3p affected the occurrence and development of osteosarcoma by targeting the SOCS1/JAK2/STAT3 signaling pathway. Therefore, miR-331-3p reduces the expression of SOCS1 by combining with its 3′UTR, thereby activating the JAK2/STAT3 signaling pathway to regulate the progression of OS. This provides a new theoretical basis for the treatment of osteosarcoma.

## 1. Introduction

Osteosarcoma (OS) is one of the most common malignant bone tumors in children and adolescents [1]. The treatment for OS mainly comprises surgical resection, systemic chemotherapy, targeted radiation therapy, immunotherapy, gene therapy, and stem cell therapy [2, 3]. Although surgical resection and neoadjuvant chemotherapy have reduced the mortality rates in surgical patients, the 5-year survival rate is still very low [4]. OS cell proliferation, migration, invasion, apoptosis, autophagy, epithelial-mesenchymal transition, and other pathophysiological processes are closely related to the development of OS [5, 6]. Unfortunately, the molecular mechanism of OS development has not yet been fully elucidated. Therefore, it is important to identify new diagnostic biomarkers and therapeutic targets.

MicroRNAs (miRNAs) are highly conserved endogenous noncoding RNAs with a length of approximately 19–25 nucleotides [7]. miRNAs are involved in many metabolic processes, including cell proliferation, differentiation, and apoptosis [8]. Many studies have confirmed that miRNA dysregulation is one of the causes of many cancers. miRNA mimics and miRNA-targeting molecules (antimiR) appear to be superior prospects for preclinical development [9]. For example, mimics of the tumor suppressor, miRNA miR-34, have entered phase I clinical trials for cancer treatment, and antimiRs targeting miR-122 have entered phase II trials for hepatitis treatment [10, 11]. Interestingly, increasing evidence supports the involvement of miR-331-3p in the occurrence and development of a variety of cancers. [12]. However, the function of miR-331-3p in OS remains unclear, and further research is needed.

Suppressor of cytokine signaling 1 (SOCS1) is a member of the SOCS family. It plays a vital role in regulating the immune system and in solving the inflammatory cascade [13, 14]. SOCS1 is also considered to be a tumor suppressor in many cancers and may act as a tumor suppressor or promoter in a manner that depends on the cell environment [15]. We aimed to determine the expression profile of miR-331-3p in osteosarcoma tissues using biological information and quantitative real-time polymerase chain reaction and to assess the effect of miR-331-3p on the growth of OS cells in vitro. We outline the potential mechanism of SOCS1 as a tumor suppressor and discuss the new evidence of SOCS1 activity as an oncogene.

## 2. Materials and Methods

### 2.1. miRNA Expression Data Analysis

Gene array expression profile data GSE39040, including 65 OS tissues and 65 paired noncancerous samples, were downloaded from the Gene Expression Omnibus database (GEO) [11]. All GEO data were analyzed in the R statistical environment. The expression values of miR-331-3p from GEO data were analyzed using the online tool, ENCORI.

### 2.2. Cell Culture and Transfection

The human OS cell lines 143b, Hos, Saos-2, U–2OS, and hFOB1.19 were obtained from the Cell Bank of the Chinese Academy of Sciences (Shanghai, China). HEK-293T cells were kindly provided by Sir Run Run Shaw Hospital, Zhejiang University School of Medicine and the Key Laboratory of Musculoskeletal System Degeneration and Regeneration Translational Research of Zhejiang Province. All cells were maintained in Dulbecco's Modified Eagle's Medium (Hyclone, LU, USA) supplemented with 10% fetal bovine serum (Gibco, NY, USA). The cells were incubated at 37°C in an atmosphere of 5% CO_2_.

miR-331-3p inhibitor or miR-331-3p mimics were transfected into 143b and Hos cells separately. Cell transfection was performed using Lipofectamine 3000 (Invitrogen, Carlsbad, CA, USA) according to the manufacturer's instructions. After transfection for 48 h, cells were harvested for further analysis.

### 2.3. RNA Extraction, Reverse Transcription, and Quantitative Real-Time PCR

Total RNA was extracted from OS cells using an Ultrapure RNA Purification Kit (Takara Bio, Shiga, Japan). Reverse transcription was performed using the Kr116 FastKing cDNA Synthesis Kit (TIANGEN, Beijing, China) according to the manufacturer's instructions. Quantitative real-time PCR (qRT-PCR) was performed in triplicate on a QuantStudio™ 3 Real-Time PCR System using SuperReal PreMix Plus ((TIANGEN, Beijing, China). miRNA quantification was determined by using Bulge-loop RT primer (RiboBio Co.Ltd, Guangdong, China). Data obtained from qPCR were analyzed using GraphPad prism. Cycle threshold (Ct) values were calculated, and the relative mRNA levels of the target genes were analyzed using the 2-ΔΔCT method. The primer sequences are shown in Table 1.

### 2.4. CCK-8 Assay

Transfected 143b and Hos cells (2 × 10^3^ cells) were cultured in a 96-well culture plate with 100 *μ*L of culture medium per well. 10 *μ*L of CCK-8 reagent (GLPBIO, CA, USA) was added to each well at 0, 24, 48, and 72 h posttreatment, followed by incubation for 3 h. The plate was then shaken on a microplate reader, a zero adjustment was performed using a blank hole, and absorbance was measured at 450 nm (optical density).

### 2.5. Wound-Healing Assay

Transfected 143b and Hos cells were cultured in serum-free medium for 24 h. A 200-*μ*L pipette tip was used perpendicular to the 6-well plate to make scratches. Photographs were taken at 0 h and 24 h and analyzed with Image J software (National Institute of Health, Bethesda, MD, USA). The cell migration rate indicated the migration and movement abilities of each group of cells. The greater the migration rate, the greater the migration and movement ability.

### 2.6. Cell Migration and Invasion Assays

Transfected 143b and Hos cells were cultured in serum-free medium for 24 h prior to migration and invasion assays. Cells (0.2 × 10^5^) in 0.2 ml of serum-free medium were seeded in the upper chamber containing 40 *μ*L of 1 mg/ml Matrigel (BD Biosciences, MA, USA), and 0.6 ml of complete medium containing 10% FBS was added to the lower chamber. After 48 h of incubation, the cells at the top of the membrane were removed with a cotton swab. The cells that migrated to the bottom well were fixed in methanol for 20 min and stained with 0.1% crystal violet solution. The number of invading cells was counted in three randomly selected optical microscope fields.

### 2.7. Colony Formation Assay

Transfected 143b and Hos cells were plated into 6-well plates and cultured for 10 days at 37°C. Colonies were then fixed with 4% paraformaldehyde for 20 min and stained with 0.1% crystal violet. The colony formation numbers (>50 cells) were counted under a microscope, and the experiment was repeated three times over.

### 2.8. Dual-Luciferase Reporter Assay

Dual-luciferase reporter plasmids were purchased from HanBio (Shanghai, China). HEK-293T cells were cultured in 6-well plates at a density of 3 × 10^4^ cells/well before transfection. Cells were then cotransfected with plasmid mixtures containing the RL reporter and FL reporter with or without the mir-331-3p 3-UTR (500 ng) and SOCS13′-UTR or negative control (NC) (10 nM final concentration) using Lipofectamine RNAiMAX (Invitrogen, AL, USA). Luciferase activity was measured using a Dual Luciferase Reporter Gene Assay Kit (Beyotime, Shanghai, China) after 48 h. For comparison, FLUC activity was normalized to RLUC activity to determine the ratio. The luciferase activity ratio of the miR-331-3p mimic group to the NC group was calculated and expressed as the fold-change.

### 2.9. Western Blot Analysis

Cells were harvested, washed, and lysed in lysis buffer (P0013, Beyotime, China) supplemented with a protease/phosphatase inhibitor cocktail. Proteins were separated using SDS-PAGE and transferred to poly vinylidene fluoride membranes (BioRad, AL, USA). The membrane was blocked overnight with 5% skim milk in Tris-buffered saline containing 0.5% Tween-20 and then incubated with primary antibodies against SOCS1 (Immunoway, USA), JAK2(Immunoway, USA), p-JAK2 (Immunoway, USA), STAT3 (Immunoway, USA), p-STAT3 (Immunoway, USA), and *β*-actin (Immunoway, USA) at 4°C. The membranes were then incubated with an HRP secondary antibodies (Dawen, HangZhou, China). Proteins were detected using an enhanced chemiluminescence kit (Beyotime, Shanghai, China) according to the manufacturer's instructions. An anti-*β*-actin antibody was used to detect uniform loading. Each experiment was performed in triplicate.

### 2.10. Subcutaneous Xenograft Tumor Models

Approximately 5 × 10^6^ 143b cells were injected subcutaneously into nude mice (female, 4 weeks old) (*n* = 6 per group) by randomization. The width and length of the tumor was measured for 4 consecutive weeks. The tumor volume was calculated as follows: volume(*mm*^3^)=(length × width^2^)/2. Mice were sacrificed 4 weeks after injection, and the tumors were collected and weighed. A part of the tumor was extracted for RNA and protein analysis, and the remaining tissue was fixed in 4% paraformaldehyde for further use.

### 2.11. Plasmid Construction and Stable Transfection

Stable transfection small interfering RNAs (siRNAs) were obtained from RiboBio (Guangzhou, China) and transfected into cells using Lipofectamine iMax (Invitrogen, AL, USA) following the manufacturer's instructions. The human lentivirus miR-331-3p sponge and the SOCS1-overexpressing lentiviral plasmid were purchased from HanBio. miRNA mimics and inhibitors were purchased from GenePharma (Shanghai, China). Transfection efficiency was verified using qRT-PCR.

### 2.12. Immunohistochemistry (IHC)

A primary antibody against SOCS1 (Immunoway, USA) was used and incubated with the cells overnight at 4°C. Thereafter, the cells were washed in phosphate-buffered saline (PBS), followed by incubation with secondary antibodies (Proteintech IL, USA) for 1 h. Finally, the cells were washed in PBS, and immunofluorescence images were captured using an inverted microscope (Nikon, Tokyo, Japan).

### 2.13. Statistical Analyses

All statistical analyses were performed using GraphPad Prism 6 software. Statistical significance was defined as a two-sided *P* value <0.05. Experiments were replicated at least three times, and the data are shown as the mean ± standard deviation (SD) in bars.

## 3. Results

### 3.1. The Expression of miR-331-3p Was Decreased in OS

**Figure 1:**
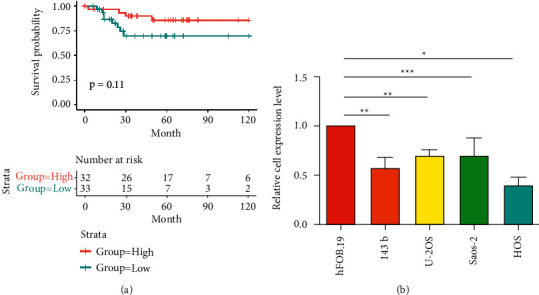
The expression of miR-331-3p decreases in OS. (a) The survival curve of the differential expression of miR-331-3p in OS. (b) qRT-PCR detects the expression level of miR-331-3p in OS cell lines. The results are presented as the mean ± standard deviation (SD) from three independent experiments. ^∗∗^*P* < 0.01.

We analyzed the expression level of miR-331-3p in osteosarcoma through bioinformatics. The analysis results showed that low expression of miR-331-3p was negatively associated with overall survival (days) in patients with OS (Figure 1(a)). Secondly, we detected the expression of miR-331-3p in U–2OS, HOS, Saos-2, 143b, and hFOB1.19 by qRT-PCR. The results indicated that miR-331-3p is downregulated in OS cell lines compared with normal cells (Figure 1(b)). These data indicate that miR-331-3p is closely related to the occurrence and development of OS.

### 3.2. MiR-331-3p Suppresses OS Cell Proliferation Metastasis and Invasion In Vitro

**Figure 2:**
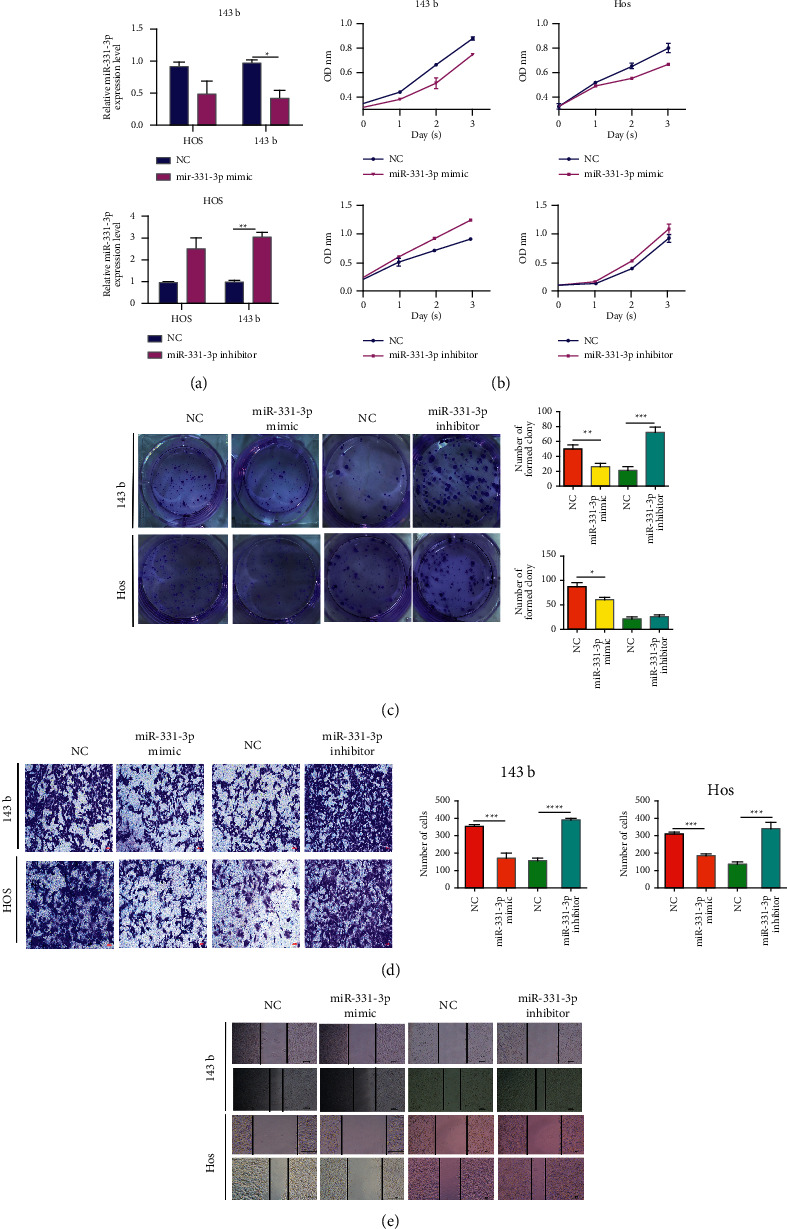
miR-331-3p can inhibit the proliferation, migration, and invasion of OS in vitro. (a) 143b and Hos are transfected with miR-331-3p mimics or inhibitors, and then cell biological functions are evaluated. (a) qRT-PCR detects the transfection efficiency of miR-331-3p. (b) The cell viability of 143b and Hos after the detection of miR-331-3p mimics/inhibitors by CCK-8. (c) Colony formation analysis of changes in OS cell proliferation ability. (d) The transwell experiment detects the invasion level of OS cells. (e) The migration ability of OS cells is analyzed by wounding experiments. The results are shown as the mean ± standard deviation (SD) from three independent experiments. ^∗∗^*P* < 0.01.

miR-331-3p acts as an oncogene in a variety of cancers, but it is rarely described in OS. Here, we evaluated the role of miR-331-3p in OS by transfecting 143b and Hos cells with miR-331-3p mimics or inhibitors. We detected transfection efficiency using qRT-PCR (Figure 2(a)). Furthermore, the CCK-8 detection and plate colony formation analysis showed that miR-331-3p over-expression further inhibited cell proliferation compared with the NC group, while miR-331-3p inhibition resulted in increased cell proliferation (Figures 2(b) and 2(c)). In addition, wound healing and transwell assays confirmed that when miR-331-3p was overexpressed, the migration and invasion ability of OS cells decreased significantly. While miR-331-3p was inhibited, 143b and Hos showed an upward trend (Figures 2(d) and 2(e)). Overall, these data indicated that miR-331-3p could inhibit the migration, invasion, and proliferation of OS cells in vitro.

### 3.3. SOCS1 is a Direct Target of miR-331-3p in OS

**Figure 3:**
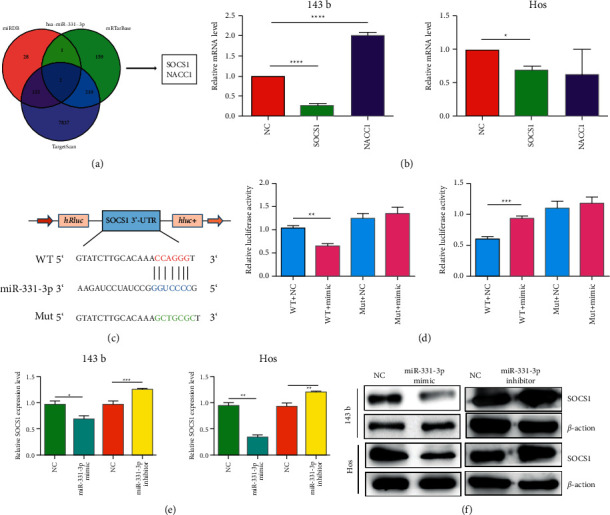
SOCS1 is a direct target of miR-331-3p and is considered an oncogene in OS. (a) Screening target genes through the intersection of three databases (TargetScan, miRDB, and miRTarBase). (b) qRT-PCR is used to evaluate the expression level of potential target genes in OS cells. (c) Schematic diagram of the binding sequence between miR-331-3p and SOCS1. (d) HEK-293T cells are transfected with miR-331-3p mimic or negative control (NC) and wild-type or mutant SOCS1^3^′-UTR, and subjected to luciferase assay. (e) qRT-PCR is used to evaluate the mRNA level of SOCS1 in OS cells transfected with miR-331-3p mimics/inhibitors. (f) Western blotting is used to evaluate the protein expression of SOCS1 in OS cells transfected with miR-331-3p mimics/inhibitors. The results are shown as the mean ± standard deviation (SD) from three independent experiments. ^∗∗^*P* < 0.01.

Prediction of SOCS1 and NACC1 as possible targets of miR-331-3p was performed by analysis of the three databases, TargetScan, miRDB, and miRTarBase (Figure 3(a)). The results showed that SOCS1 had the greatest promotion effect on cell proliferation by qRT-PCR (Figure 3(b)); therefore, it was selected for further research. The results of the dual luciferase reporter assay verified the targeting relationship between miR-331-3p and SOCS1 (Figures 3(c) and 3(d)). In addition, the results of RT-qPCR and Western blot analysis showed that the expression of SOCS1 in OS cells was reversely regulated by miR-331-3p mimics or inhibitors (Figures 3(e) and 3(f)). This indicates that SOCS1 is a direct target of miR-331-3p.

It has been reported that SOCS1 acts as an oncogene in various cancers, but its role in OS is unclear [15]. To determine whether the expression of endogenous SOCS1 is closely related to the pathogenesis of OS, 143b and Hos cells were transfected with si- SOCS1 1 to evaluate their functions in vitro. Transfection efficiency was verified by qRT-PCR (Figure S1A). Wound healing and transwell assays showed that lower levels of SOCS1 inhibited OS cell metastasis and invasion (Figures S1B and S1C). On the other hand, compared with the control group, OS cells with downregulated SOCS1 expression had a lower growth rate and reduced colony formation (Figure S1D). The CCK-8 results showed that lower levels of SOCS1 significantly inhibited cell proliferation (Figure S1E). In summary, our results indicate that over-expression of miR-331-3p may inhibit the progression of OS through SOCS1.

### 3.4. miR-331-3p Promotes OS Progression via SOCS1

**Figure 4:**
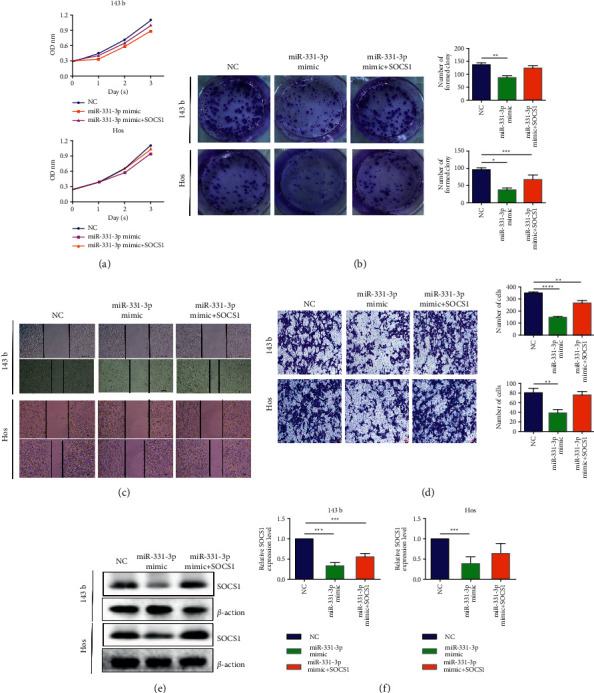
SOCS1 regulates the tumor suppressor function of miR-331-3p in OS cells in vitro. (a) CCK-8 assay shows that miR-331-3p over-expression inhibits the effect of SOCS1 silencing on cell growth. (b) Colony formation analysis of changes in OS cell proliferation ability. (c) Wound repair experiments show the effect of SOCS1 over-expression on migration ability. (d) The invasion ability of cells after cotransfection with miR-331-3p and SOCS1 is determined by the invasion test. (e) Western blot is used to detect the protein expression level of SOCS1 in OS cells after miR-331-3p alone transfection and miR-331-3p and SOCS1 cotransfection. (f) Detection of SOCS1 mRNA expression level in OS cells after miR-331-3p alone transfection and miR-331-3p and SOCS1 cotransfection by quantitative real-time polymerase chain reaction (qRT-PCR). The results are shown as the mean ± standard deviation (SD) from three independent experiments. ^∗∗^*P* < 0.01.

The SOCS1 over-expression plasmid was constructed and transfected into 143b and Hos cells with stable over-expression of miR-331-3p to further investigate whether miR-331-3p affects OS progression by targeting SOCS1. The increased cell viability (Figure 4(a)) and proliferation (Figure 4(b)) capabilities of OS cells by miR-331-3p over-expression were all abolished by SOCS1 over-expression. In addition, it was found that high expression of SOCS1 can rescue the downregulation of miR-331-3p-deficient cells through cell migration and transwell experiments (Figures 4(c) and 4(d)). Protein and gene levels also verified the above phenomenon (Figures 4(e) and 4(f)). Taken together, these findings indicate that miR-331-3p is involved in OS progression by targeting SOCS1.

### 3.5. miR-331-3p Regulates the Biological Functions of OS through the SOCS1/JAK2/STAT3 Pathway

**Figure 5:**
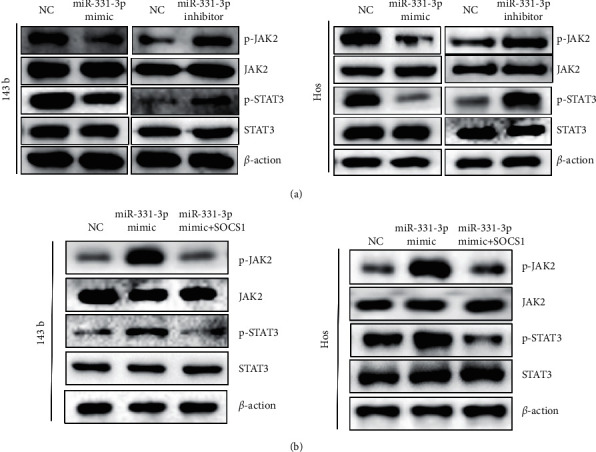
miR-331-3p inhibits SOCS1 leading to activation of the JAK2/STAT3 pathway. (a) OS cells transfected with miR-331-3p mimic/inhibitor. Protein expressions of JAK2, p-JAK2, STAT3, and p-STAT3 were examined by Western blot. (b) OS cells transfected with NC or miR-331-3p minic or cotransfected with miR-331-3p minic and the SOCS1 over-expression plasmid. Protein expressions of JAK2, p-JAK2, STAT3, and p-STAT3 were examined by Western blot.

Next, we further explored the underlying molecular mechanisms of miR-331-3p. According to the reports, miR-221-3p in M2-TAM exosomes aggravated the progression of OS by regulating the SOCS3/JAK2/STAT3 axis [16]. We found that miRNA-331-3p activates the JAK2/STAT3 phosphorylation level by inhibiting SOCS1 (Figures 5(a) and 5(b)). These results suggest that miRNA-331-3p may act through the SOCS1/JAK2/STAT3 pathway.

### 3.6. SOCS1 Acts as a Sponge for miR-331-3p to Promote Tumorigenesis In Vivo

**Figure 6:**
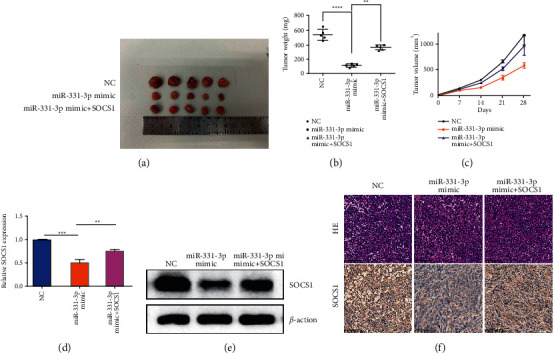
SOCS1 acts as a target of miR-331-3p to promote tumorigenesis in vivo. 143b cells are inoculated subcutaneously in female nude mice at a density of 5 × 10^6^ cells. (a) After 4 weeks, the tumor is dissected and imaged. (b) Average tumor weight of mice. The data represents the mean ± standard error of the mean (SEM) (*n* = 5 per group). (c) Tumor volume (ab^2^/2) was recorded every seven days after mice were injected with stable OS cells. The data represents the mean ± SEM (*n* = 5 per group). (d) qRT-PCR analysis of SOCS1 mRNA expression in xenograft mouse tumors. (*n* = 5 in each group). (e) Western blot analysis of SOCS1 protein expression levels in different groups of tumors. (f) HE and immunohistochemistry staining shows the tumor structure and relative protein level of SOCS1 in tumors. The results are shown as the mean ± standard deviation (SD) from three independent experiments. ^∗∗^*P* < 0.01.

An over-expression/silencing SOCS1 lentiviral plasmid was constructed and transfected into OS cells. The stabilized cells were used to construct a mouse orthotopic xenograft model to determine whether miR-331-3p and SOCS1 also function in vivo. The tumor volume of the miR-331-3p over-expression group was significantly smaller than that of the control group, while the cotransfection group showed a reversed trend (Figure 6(a)). The tumor weights of the three groups also showed the same difference (Figure 6(b)). The upregulated expression of miR-331-3p also suppressed tumor volume in subcutaneous xenograft models (Figure 6(c)). In addition, miR-331-3p mimics significantly reduced SOCS1 mRNA expression levels, and the cotransfection group showed a reversed trend (Figure 6(d)). Western blot results indicate that miR-331-3p affects the occurrence and development of osteosarcoma through the SOCS1/JAK2/STAT3 pathway (Figure 6(e)). The results of IHC showed that the average immunopositive area of SOCS1 decreased under the influence of miR-331-3p, and SOCS1 offset this change again (Figure 6(f)). These results indicate that miR-331-3p may participate in processes such as OS growth and metastasis through SOCS1.

## 4. Discussion

miRNAs are important regulators of gene expression and are involved in a variety of cellular physiological processes, such as cell growth, movement, and differentiation [17]. Nowadays, more and more evidence has confirmed that miRNAs play a key role in cancer malignancies by acting as tumor suppressor genes or oncogenes. Therefore, miRNA can be used as an important indicator of prognosis and treatment [18]. miR-331-3p is a newly discovered miRNA that has been confirmed as a tumor suppressor, and it is usually downregulated in different cancers. For example, miR-331-3p is significantly downregulated in triple-negative breast cancer (TNBC) tissues and cell lines. One study reported that over-expression of miR-331-3p inhibits the increase in proliferation and apoptosis of TNBC cells [19]. The expression of miRNA-331-3p in clinical specimens and cells of nasopharyngeal carcinoma was also significantly reduced, while the over-expression of miRNA-331-3p inhibited cell proliferation and invasion. In addition, over-expression of miRNA-331-3p reduced the expression of the target gene elF4B, thereby inhibiting phosphorylation of phosphoinositide 3-kinase and serine/threonine kinase [20]. However, little is known about the biological functions of miR-331-3p in OS.

In this study, we used bioinformatics to analyze GEO and TCGA data and found that low levels of miR-331-3p are closely related to overall survival rate and are an independent prognostic factor affecting the overall survival rate of patients. The qRT-PCR results showed that the expression of miR-331-3p was significantly downregulated in the OS cell line, confirming this conclusion. Functional tests showed that the over-expression of miR-331-3p inhibited the metastasis and invasion ability of OS cells in vitro and reduced cell viability and proliferation ability, whereas the knockdown of miR-331-3p promotes cell migration and invasion. Our results indicate that miR-331-3p is an inhibitory miRNA in OS cells. This is consistent with previous studies of other cancers.

**Figure 7:**
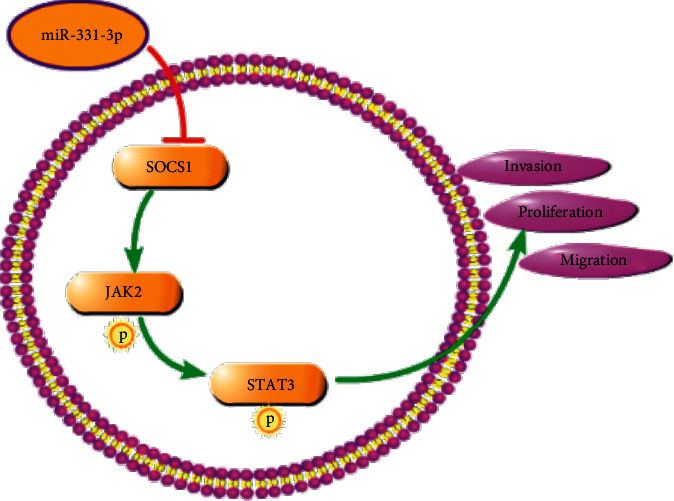
Graphical abstract. miR-331-3p targets the inhibition of SOCS1, thereby activating the phosphorylation level of JAK2/STAT3, and affecting the proliferation, migration, and invasion of OS cells. The arrow indicates activation, and the T symbol indicates inhibition.

SOCS1, also known as STAT-induced STAT inhibitor (SSI) or JAK binding protein (JAB), is a member of the SOCS family [21]. SOCS1 negatively regulates cytokines by activating the JAK-STAT signaling pathway [22]. According to literature reports, m6A mRNA controls T cell methylation by targeting the IL-7/STAT5/SOCS pathway [23]. The HuoXueTongFu formula alleviates intraperitoneal adhesion by regulating macrophage polarization and the SOCS/JAK2/STAT/PPAR- *γ* signaling pathway [24]. Recent studies have shown that SOCS1 can play a role in cancer by regulating oncogenic signal transduction pathways. Zhang et al. found that SOCS5 can inhibit HCC cell migration and invasion in vitro by activating PI3K/Akt/mTOR-mediated autophagy [25]. Here, through the analysis of the three databases—TargetScan, miRDB, and miRTarBase—we speculate that SOCS1 is the target gene of miR-331-3p. The dual luciferase results confirmed that SOCS1 was the target effector of miR-331-3p. The rescue experiment once again verified that miR-331-3p inhibits the occurrence of OS cells and tumors by regulating SOCS1. Next, we investigated other possible miR-331-3p/SOCS1 regulatory pathways and found, through Western blotting, that miR-331-3p activates the JAK2/STAT3 pathway by inhibiting SOCS1, thereby affecting the progress of OS (Figure 7).

## 5. Conclusion

In conclusion, our results confirm that miR-331-3p inhibits the proliferation, metastasis, and invasion of osteosarcoma cells. Further mechanistic studies found that miR-331-3p participates in targeting SOCS1 and upregulates JAK2/STAT3 to affect the progression of OS. The molecular mechanism explored in this study may provide a new theoretical basis for the pathogenesis and treatment of osteosarcoma.

## Figures and Tables

**Table 1:** Primer sequence.

The target	Forward (5′–3′)	Reverse (5′–3′)
SOCS1	CCAGGTGGCAGCCGACAATG	CGAGGAGGAGGAAGAGGAGGAGA
GAPDH	TGGTTGAGCACAGGGTACTT	CCAAGGAGTAAGACCCCTGG

## Data Availability

The figure data used to support the findings of this study are included within the article and Supplementary Materials.

## Abbreviations

OS: Osteosarcoma
miRNAs: MicroRNAs
SOCS1: Suppressor of cytokine signaling 1
NC: Negative control
siRNAs: Small interfering RNAs
IHC: Immunohistochemistry
PBS: Phosphate-buffered saline
TNBC: Triple-negative breast cancer
FBS: Fetal bovine serum.

## Supplementary Materials

Supplementary Materials Figure S1: miR-331-3p inhibit the progression of OS through SOCS1. (A) qRT-PCR to detect the transfection efficiency of siSOCS1 in osteosarcoma. (B) The migration ability of OS cells is analyzed by wounding experiment. (C) The transwell experiment detects the invasion level of OS cells. (D) Colony formation analysis of changes in OS cell proliferation ability. (E) The cell viability of 143b and Hos after the detection of siSOCS1 by CCK-8.

## References

[1] Wittig J. C., Bickels J., Priebat D. (2002). Osteosarcoma: a multidisciplinary approach to diagnosis and treatment. *American Family Physician*.

[2] ElKordy M. A., ElBaradie T. S., ElSebai H. I., Amin A. A. E., KhairAlla S. M. (2018). Osteosarcoma of the jaw: challenges in the diagnosis and treatment. *Journal of the Egyptian National Cancer Institute*.

[3] Wang J., Liu S., Shi J. (2019). The role of miRNA in the diagnosis, prognosis, and treatment of osteosarcoma. *Cancer Biotherapy and Radiopharmaceuticals*.

[4] Reed D. R., Hingorani P., Anderson P. M. (2020). Relapsed osteosarcoma trial concepts to match the complexity of the disease. *Advances in Experimental Medicine and Biology*.

[5] Cersosimo F., Lonardi S., Bernardini G. (2020). Tumor-associated macrophages in osteosarcoma: from mechanisms to therapy. *International Journal of Molecular Sciences*.

[6] Camuzard O., Santucci-Darmanin S., Carle G. F., Pierrefite-Carle V. (2019). Role of autophagy in osteosarcoma. *Journal of Bone Oncology*.

[7] Lu T. X., Rothenberg M. E. (2018). MicroRNA. *The Journal of Allergy and Clinical Immunology*.

[8] Giza D. E., Calin G. A. (2015). microRNA and chronic lymphocytic leukemia. *Advances in Experimental Medicine and Biology*.

[9] Iorio M. V., Croce C. M. (2012). MicroRNA dysregulation in cancer: diagnostics, monitoring and therapeutics. A comprehensive review. *EMBO Molecular Medicine*.

[10] Zhang L., Liao Y., Tang L. (2019). MicroRNA-34 family: a potential tumor suppressor and therapeutic candidate in cancer. *Journal of Experimental & Clinical Cancer Research*.

[11] Rupaimoole R., Slack F. J. (2017). MicroRNA therapeutics: towards a new era for the management of cancer and other diseases. *Nature Reviews Drug Discovery*.

[12] Chen T., Ma L., Cui J., Geng J., Zeng Y., Chen W. (2019). Construction of miR-331-3p overexpression vector and its effect on cell proliferation. *Sheng Wu Gong Cheng Xue Bao*.

[13] He C., Yu C.-R., Mattapallil M. J., Sun L., Larkin III J., Egwuagu C. E. (2016). SOCS1 mimetic peptide suppresses chronic intraocular inflammatory disease (uveitis). *Mediators Inflamm*.

[14] Ilangumaran S., Bobbala D., Ramanathan S. (2017). SOCS1: regulator of T cells in autoimmunity and cancer. *Current Topics in Microbiology and Immunology*.

[15] Beaurivage C., Champagne A., Tobelaim W. S., Pomerleau V., Menendez A., Saucier C. (2016). SOCS1 in cancer: an oncogene and a tumor suppressor. *Cytokine*.

[16] Quero L., Tiaden A. N., Hanser E. (2019). miR-221-3p drives the shift of M2-macrophages to a pro-inflammatory function by suppressing JAK3/STAT3 activation. *Frontiers in Immunology*.

[17] Saliminejad K., Khorshid H. R. K., Fard S. S., Ghaffari S. H. (2019). An overview of microRNAs: biology, functions, therapeutics, and analysis methods. *Journal of Cellular Physiology*.

[18] Shah V., Shah J. (2020). Recent trends in targeting miRNAs for cancer therapy. *Journal of Pharmacy and Pharmacology*.

[19] Zhao M., Zhang M., Tao Z., Cao J., Wang L., Hu X. (2020). miR-331-3p suppresses cell proliferation in TNBC cells by downregulating NRP2. *Technology in Cancer Research and Treatment*.

[20] Xuefang Z., Ruinian Z., Liji J. (2020). miR-331-3p inhibits proliferation and promotes apoptosis of nasopharyngeal carcinoma cells by targeting elf4B-PI3K-akt pathway. *Technology in Cancer Research and Treatment*.

[21] Sharma J., Larkin J. (2019). Therapeutic implication of SOCS1 modulation in the treatment of autoimmunity and cancer. *Frontiers in Pharmacology*.

[22] Durham G. A., Williams J. J., Nasim M. T., Palmer T. M. (2019). Targeting SOCS proteins to control JAK-STAT signalling in disease. *Trends in Pharmacological Sciences*.

[23] Li H. B., Tong J., Zhu S. (2017). m 6 A mRNA methylation controls T cell homeostasis by targeting the IL-7/STAT5/SOCS pathways. *Nature*.

[24] Zhao M., Bian Y.-Y., Yang L.-L. (2019). HuoXueTongFu Formula Alleviates Intraperitoneal Adhesion by Regulating Macrophage Polarization and the SOCS/JAK2/STAT/PPAR-gamma Signalling Pathway. *Mediators Inflamm*.

[25] Zhang M., Liu S., Chua M. S. (2019). SOCS5 inhibition induces autophagy to impair metastasis in hepatocellular carcinoma cells via the PI3K/Akt/mTOR pathway. *Cell Death & Disease*.

[26] Zu D., Dong Q., Chen S. (2021). miRNA-331-3p affects the proliferation, metastasis and invasion of osteosarcoma through SOCS1/JAK2/STAT3. https://assets.researchsquare.com/files/rs-1190352/v1/5c32f96d-f0ee-4b98-90b7-c76158b2f1a0.pdf?c=1640878987.

