# Supplementary material for: miRNA-331-3p Affects the Proliferation, Metastasis, and Invasion of Osteosarcoma through SOCS1/JAK2/STAT3
Source: J Oncol. 2022 Sep 26;2022:6459029. doi: 10.1155/2022/6459029 (PMC9529391; doi:10.1155/2022/6459029)
Supplement: Supplementary Materials — Figure S1: miR-331-3p inhibit the progression of OS through SOCS1. (A) qRT-PCR to detect the transfection efficiency of siSOCS1 in osteosarcoma. (B) The migration ability of OS cells is analyzed by wounding experiment. (C) The transwell experiment detects the invasion level of OS cells. (D) Colony formation analysis of changes in OS cell proliferation ability. (E) The cell viability of 143b and Hos after the detection of siSOCS1 by CCK-8. [file 6459029.f1.docx]

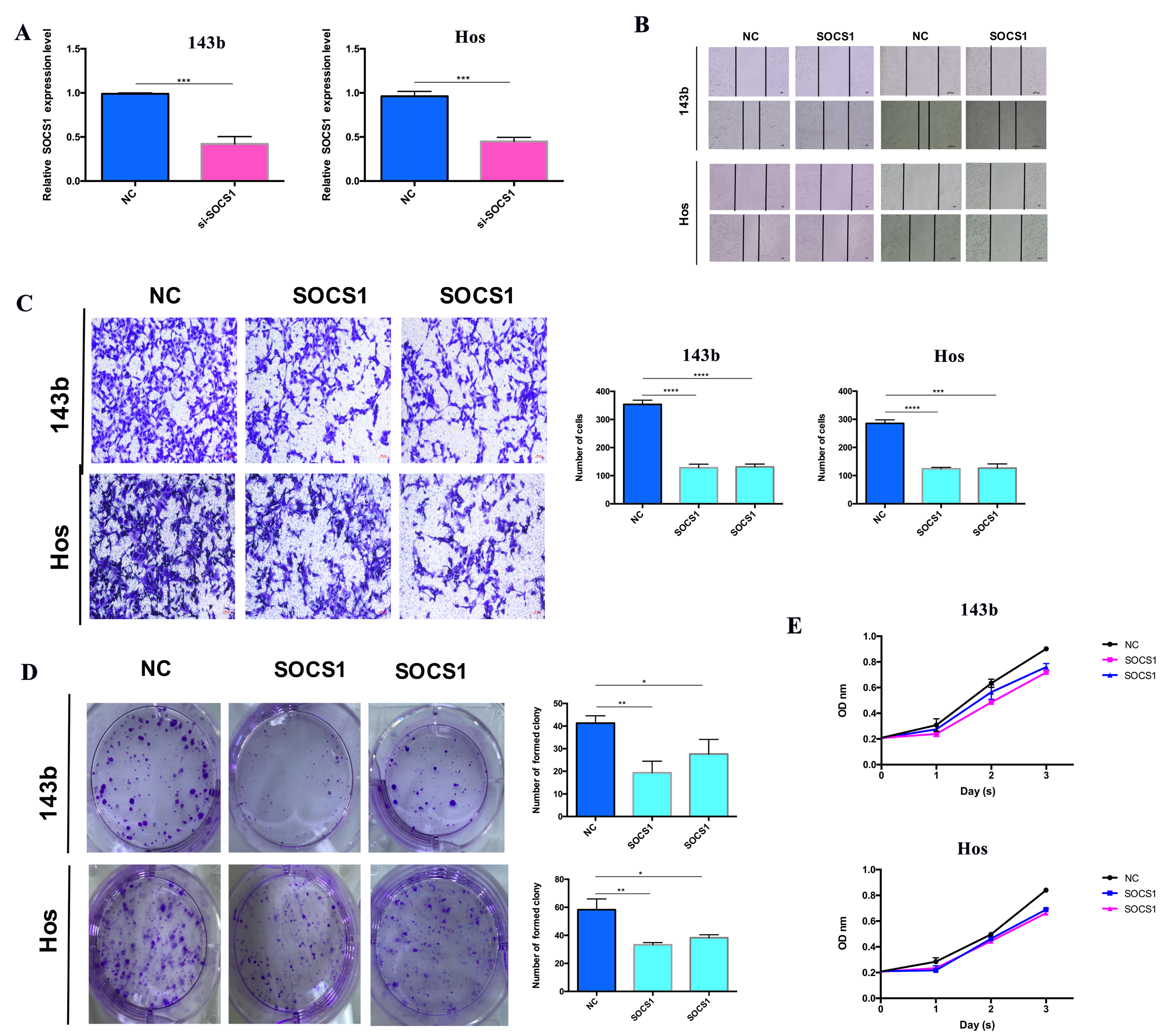


FIGURE S1: miR-331-3p inhibit the progression of OS through SOCS1. (A) qRT-PCR to detect the transfection efficiency of siSOCS1 in osteosarcoma. (B) The migration ability of OS cells is analyzed by wounding experiment. (C) The transwell experiment detects the invasion level of OS cells. (D) Colony formation analysis of changes in OS cell proliferation ability. (E) The cell viability of 143b and Hos after the detection of siSOCS1 by CCK-8.
